# Visor Flap-Assisted Segmental Mandibulectomy and Immediate Free Fibula Reconstruction for Extensive Plexiform Ameloblastoma of the Anterior Mandible: A Case Report

**DOI:** 10.7759/cureus.116344

**Published:** 2026-09-16

**Authors:** Yaseer Arafat, Pradeep Christopher, Sayantan Bhattacharya, Sharmistha Chakraborty

**Affiliations:** 1 Dentistry, Orangeclinics, Chennai, IND; 2 Oral and Maxillofacial Surgery, Thai Moogambigai Dental College and Hospital, Chennai, IND

**Keywords:** ameloblastoma, fibula, intraosseous, mandible, plexiform

## Abstract

Ameloblastoma is a benign but locally aggressive odontogenic tumour commonly affecting the mandible. This case report describes the surgical management of mandibular ameloblastoma in a 65-year-old male with a gradually enlarging anterior mandibular swelling for two years. Clinical and radiological evaluation revealed an expansile lesion in the symphyseal region, and histopathology confirmed plexiform ameloblastoma.

Considering the lesion’s extent, segmental mandibulectomy was performed using a visor flap and pull-through approach. Immediate reconstruction was achieved with a pre-bent 2.5-mm titanium reconstruction plate and a free fibular flap with microvascular anastomosis. Postoperative recovery was satisfactory, restoring mandibular continuity, function, and facial aesthetics.

## Introduction

Ameloblastoma is a benign but locally aggressive odontogenic tumour arising from the epithelial components of the tooth-forming apparatus. The term was introduced by Ivy and Churchill in 1934 to describe a tumour whose cells resemble ameloblasts involved in enamel formation [1]. Later, Robinson [2] characterised ameloblastoma as a non-functional, unicentric tumour with intermittent growth that is anatomically benign but clinically persistent due to its infiltrative nature and tendency to recur. According to the World Health Organization classification [3], ameloblastoma represents a true neoplasm derived from enamel organ-type epithelium that does not differentiate to the point of enamel formation.

Ameloblastoma accounts for approximately 19-40% of all odontogenic tumours and most frequently involves the posterior mandible [4], particularly the molar-ramus region. However, lesions may also occur in the anterior mandible and maxilla [4]. The tumour typically presents during the second to fourth decades of life, although it may occur across a wide age range. Clinically, patients usually present with a painless, slowly enlarging swelling of the jaw, which may lead to facial asymmetry, tooth displacement, mobility, root resorption, and occasionally, paraesthesia or nasal obstruction [5]. Radiographically, ameloblastoma commonly appears as a well-defined unilocular or multilocular radiolucent lesion with the classic “soap-bubble” or “honeycomb” appearance.

Histopathologically, several variants have been described, including follicular, plexiform, acanthomatous, granular cell, basal cell, and desmoplastic types [6]. These subtypes exhibit varying biological behaviour and recurrence potential [7]. Because of its locally invasive nature and high recurrence rate following conservative treatment, surgical resection with adequate margins is often recommended for extensive lesions. Historically, major symphyseal resections have utilised a midline lip-split approach. Although a lip-split provides direct visualisation, it carries notable drawbacks, including permanent facial scarring, lower lip asymmetry, potential mental nerve morbidity, and lip incompetence, which may lead to chronic drooling. To overcome these morbidities, the visor flap with a pull-through technique offers an alternative approach for anterior arch lesions. This approach provides encapsulated 3D surgical exposure of the symphyseal tumour while leaving the facial skin and lip continuity completely intact.

Reconstruction of mandibular defects following tumour excision is essential to restore facial aesthetics, mandibular continuity, and oral function. This case report describes the surgical management of a mandibular ameloblastoma and its reconstruction using contemporary reconstructive techniques [8]. We demonstrate the utility of combining a visor flap pull-through approach with precontoured rigid fixation and an immediate microvascular-free fibula flap to achieve complete oncological ablation while eliminating external facial scarring and maintaining facial function.

## Case presentation

A 65-year-old man presented to the Department of Oral and Maxillofacial Surgery, Thaimoogambigai Dental College and Hospital, Chennai, with a chief complaint of swelling associated with mild pain in the anterior region of the mandible for the past two years. The patient had been apparently asymptomatic two years prior, after which he noticed a swelling in the anterior part of the lower jaw that gradually increased in size. The swelling was associated with mild pain. The patient had no significant past medical history.

On extraoral examination, a diffuse swelling was noted in the anterior mandibular region involving the symphysis and parasymphysis, resulting in facial asymmetry (Figure 1a). The swelling appeared roughly oval in shape with ill-defined margins and extended across the lower anterior border of the mandible (Figure 1b). The overlying skin appeared normal, with no evidence of ulceration, sinus discharge, or other secondary changes. Mild fullness and expansion of the chin region were evident on both frontal and worm’s-eye views. No visible cervical lymphadenopathy or scar was noted. The patient’s mouth opening appeared adequate, and no deviation of the mandible was observed during opening. Temporomandibular joint movements were within normal limits.

**Figure 1:**
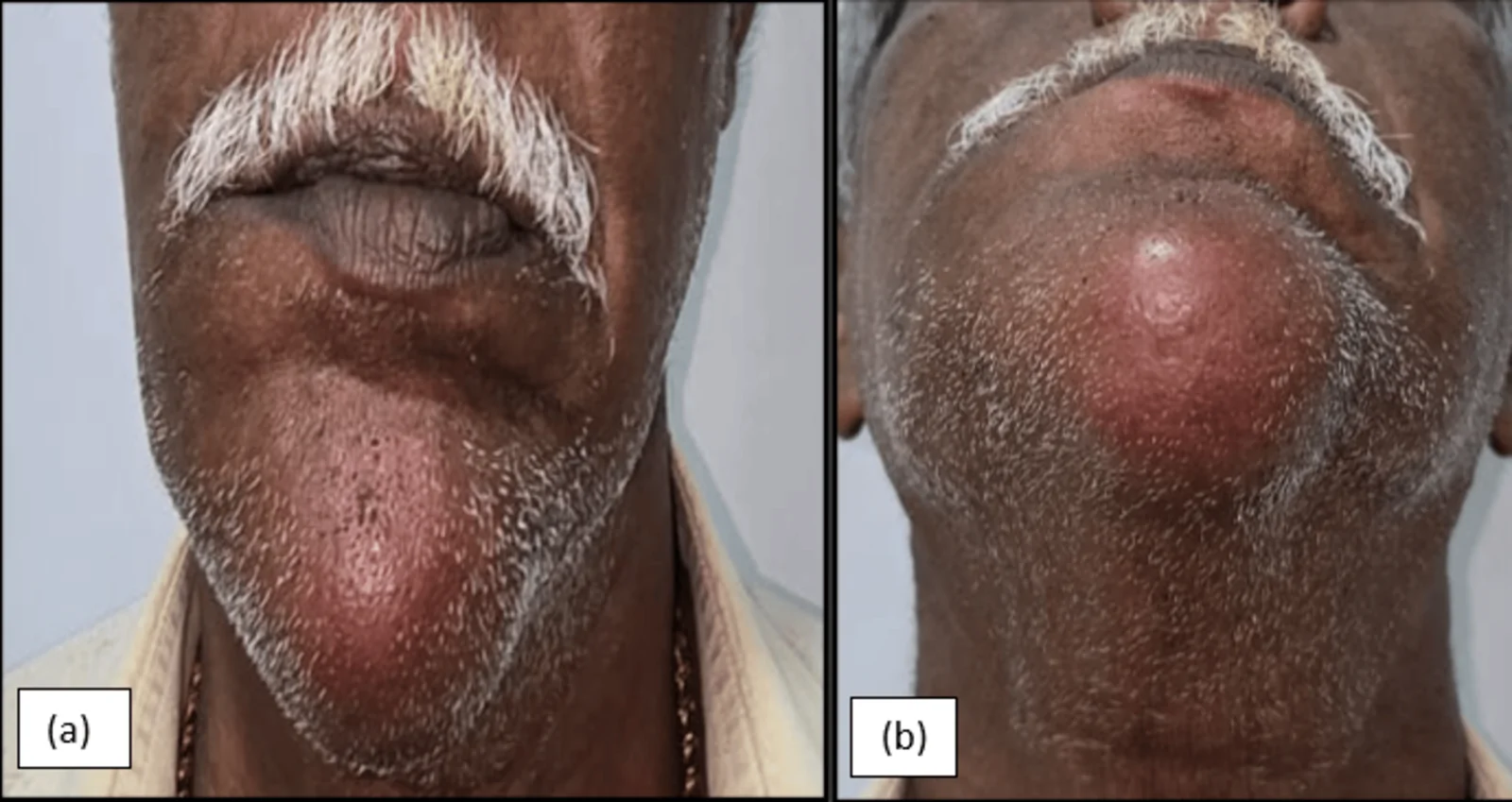
Extraoral image of the lesion: (a) frontal view; (b) worm’s eye view.

On intraoral examination, both the mandible and maxilla were edentulous. A diffuse swelling was present in the anterior mandibular alveolar region involving the symphysis, with obliteration of the labial vestibule (Figure 2). The overlying mucosa appeared stretched and smooth, with mild erythema but no ulceration or discharge. Cortical expansion was evident in the anterior mandible. No obvious mucosal perforation, bleeding, or pus discharge was noted.

**Figure 2:**
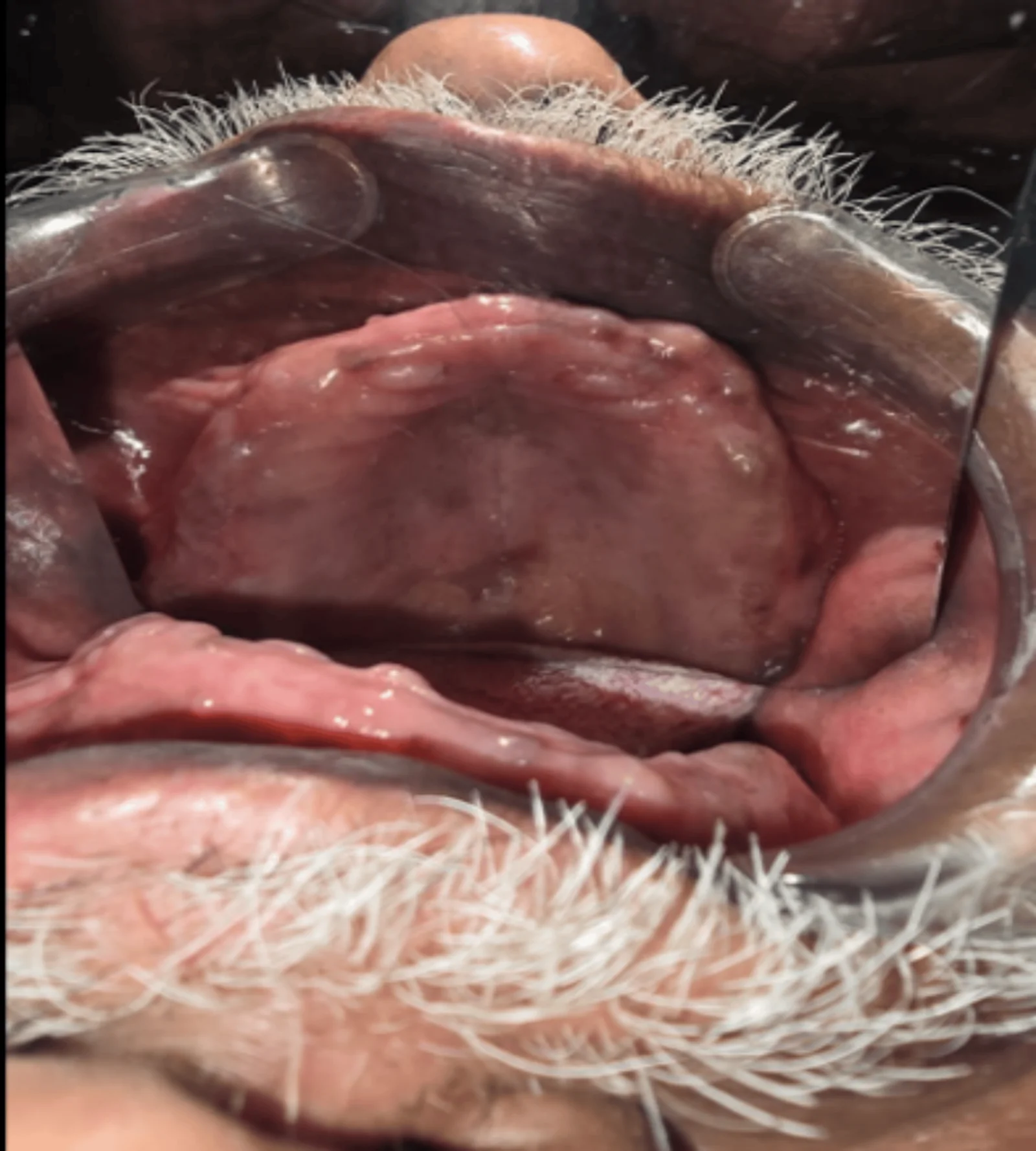
Intraoral picture showing diffuse swelling at the anterior mandibular alveolar region.

A CT scan with 3D reconstruction revealed a large expansile osteolytic lesion involving the anterior mandible and crossing the midline, with marked cortical thinning and destruction involving the symphysis and parasymphysis regions. The lesion measured approximately 6.5 × 4.0 × 3.5 cm in anteroposterior, superoinferior, and buccolingual dimensions, respectively. It also demonstrated extensive bony expansion and perforation of the cortical plates, suggestive of an aggressive odontogenic lesion with soft tissue extension, consistent with ameloblastoma (Figure 3).

**Figure 3:**
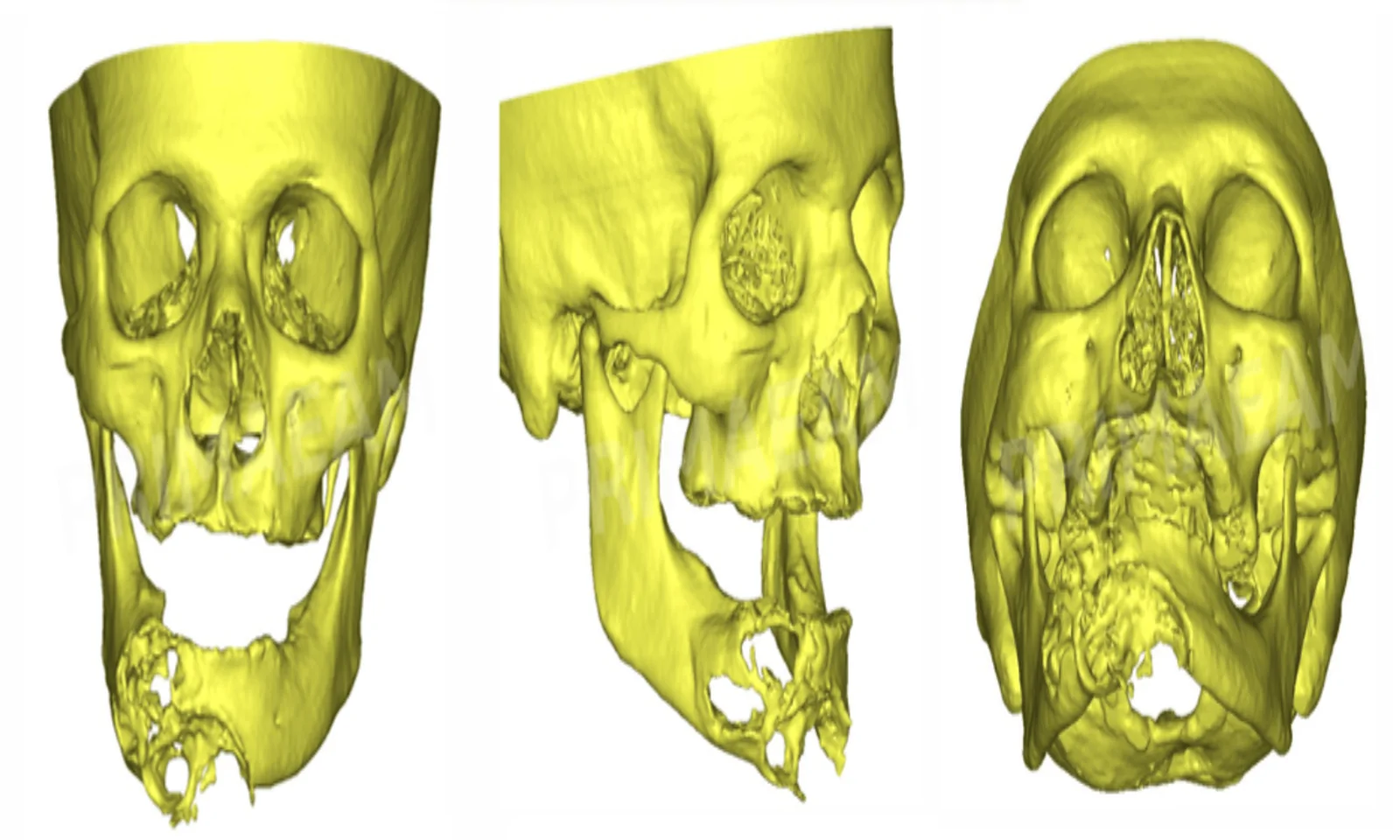
3D reconstructed images of CT scan for precise surgical planning showing the location of the lesion in mandible.

An incisional biopsy was performed under local anaesthesia on a representative area of the lesion, and the specimen was sent for histopathological examination. Molecular/immunohistochemical analyses were not performed in this case because the required facilities were not available at our centre. Based on the clinical and radiographic findings, a provisional diagnosis of intraosseous ameloblastoma was made, which was subsequently confirmed histopathologically as a plexiform type of ameloblastoma (Figure 4). To ensure complete oncological clearance with negative margins, a radical segmented mandibulectomy was planned, extending from the right mandibular canine to the left mandibular canine.

**Figure 4:**
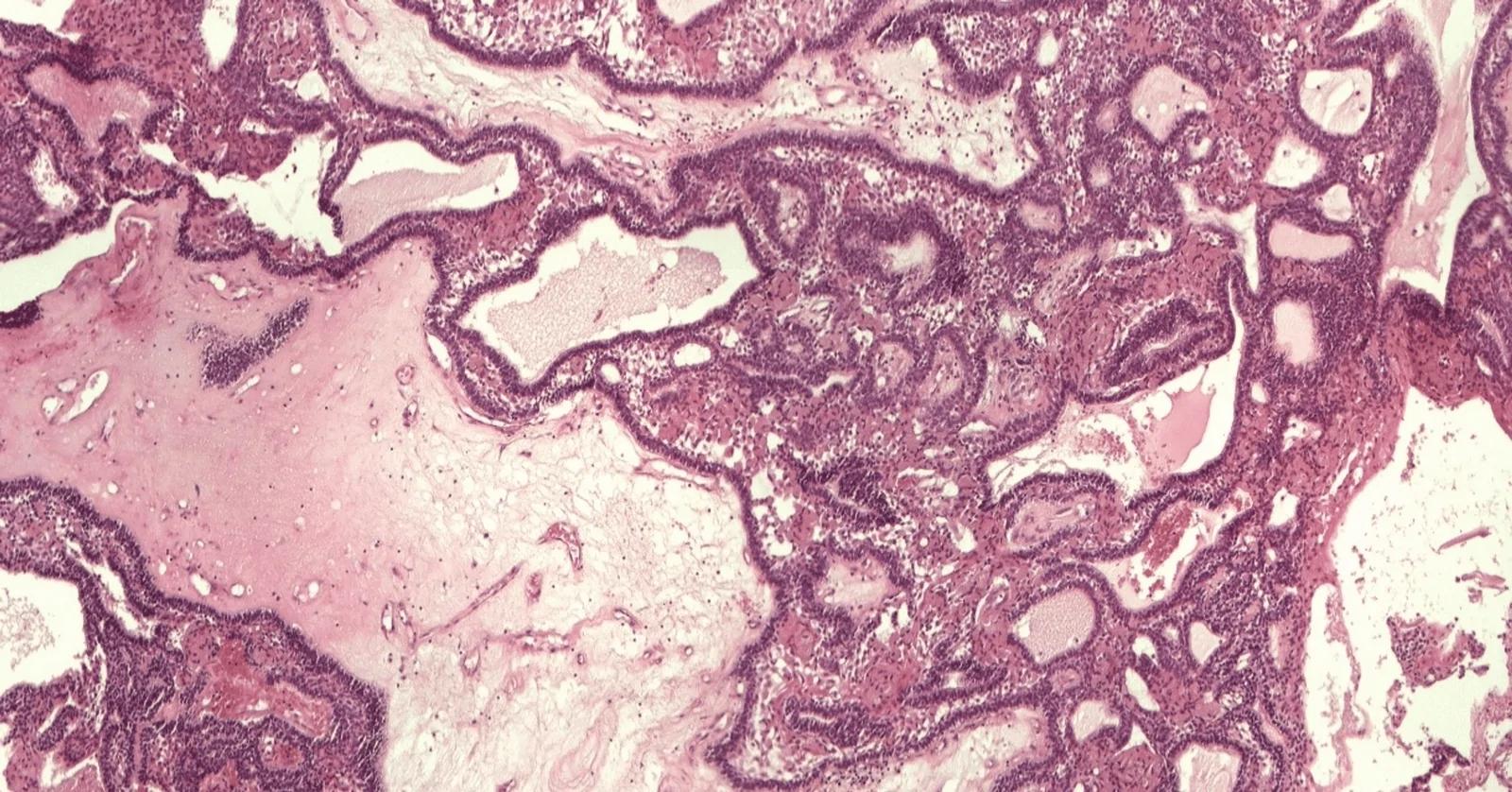
Histopathological section showing anastomosing and interconnected epithelial cords and islands in a characteristic plexiform pattern, with peripheral columnar ameloblastoma-like cells demonstrating nuclear palisading and centrally located, loosely arranged stellate-reticulum-like cells within a fibrous/myxoid stroma.

Surgery was planned under general anaesthesia after a proper evaluation of the patient's systemic status and consultation with an anaesthesiologist. Under general anaesthesia with nasotracheal intubation, the patient was prepared and draped in a standard sterile manner for a major head and neck procedure. To access the pathology while avoiding a midline lip-split incision, a visor flap with a pull-through approach was selected. A visor incision line was marked (Figure 5a). The incision was carried through the skin, subcutaneous tissue, and platysma. The flap was elevated superiorly over the lower border of the mandible to achieve an encapsulated exposure of the tumour projecting in 3D over the symphysis region (Figures 5b, 5c).

**Figure 5:**
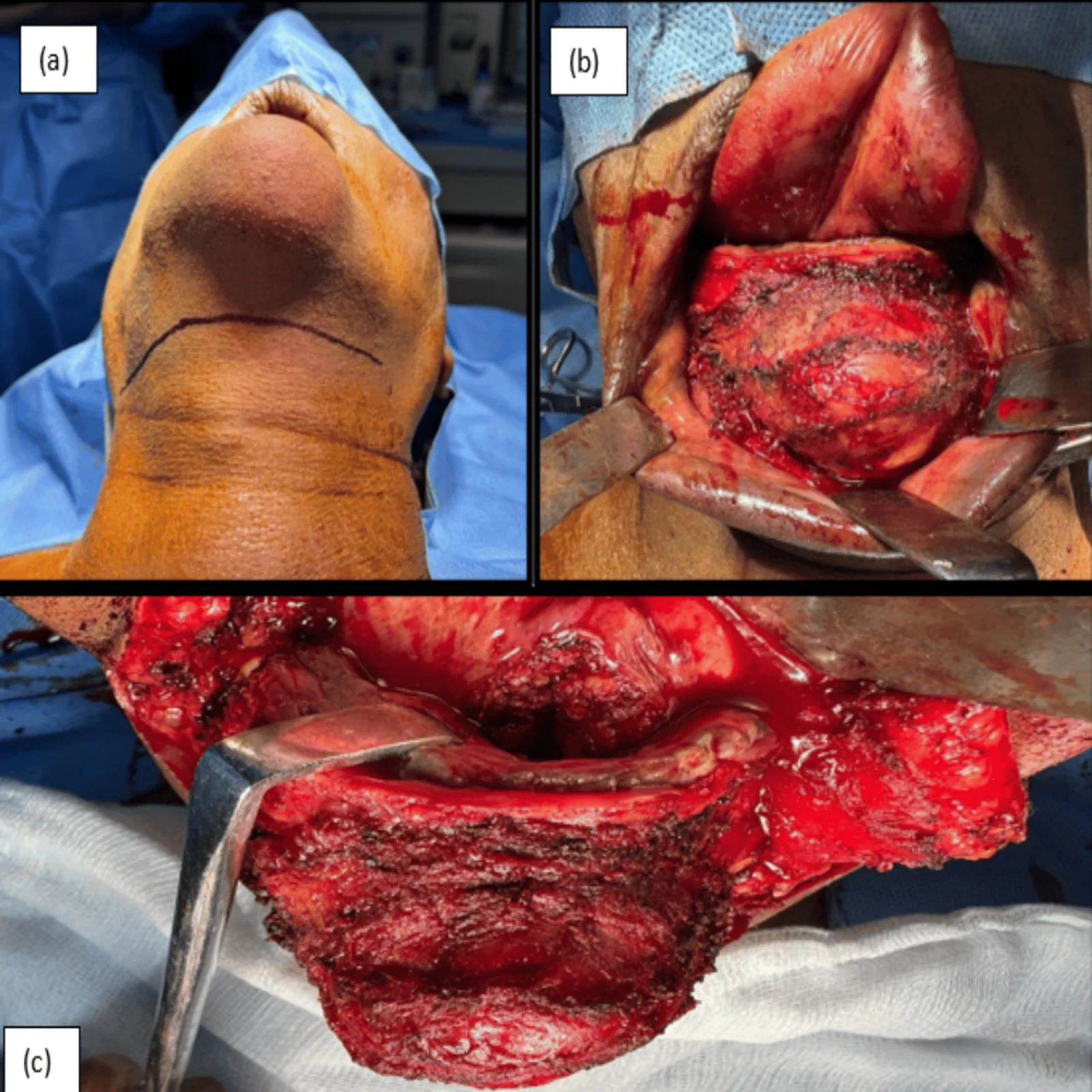
(a) Image showing visor incision marking on the neck; (b) visor flap, pull-through approach to expose the surgical site; (c) bird’s eye view of the tumour.

To maintain precise anatomical relationships post-resection, pre-bending of a 2.5-mm titanium reconstruction plate was performed along the native lower border of the mandible prior to any bone cuts. It was temporarily secured by fixing three holes on either side of the planned osteotomy line with temporary screws to maintain the maximum mandibular relationship, which was perfectly locked in before tumour ablation. Following segmental mandibulectomy, the plate was repositioned and definitively fixed using the same predrilled holes, thereby facilitating reproduction of the pre-resection mandibular contour and condylar position and preservation of the pre-existing vertical dimension.

The tumour mass, along with the involved symphyseal segment of the mandible and a safe margin of 1.5 cm, was completely excised en bloc (Figure 6a). The mental nerves were preserved bilaterally during the resection, and the patient subsequently demonstrated intact sensation over the lower lip and chin, without clinically evident paraesthesia or numbness. Concurrently, an elective neck dissection encompassing levels Ia, Ib, IIa, and IIb was performed bilaterally to clear the regional lymph node basins because of significant soft-tissue involvement and tumour spillage into the operative field, following which primary reconstruction was performed with the precontoured titanium reconstruction plate (Figure 6b). Intraoperative frozen-section analysis was not performed because this facility was not available at our centre, and the additional cost and logistical requirements associated with obtaining this service were not feasible.

**Figure 6:**
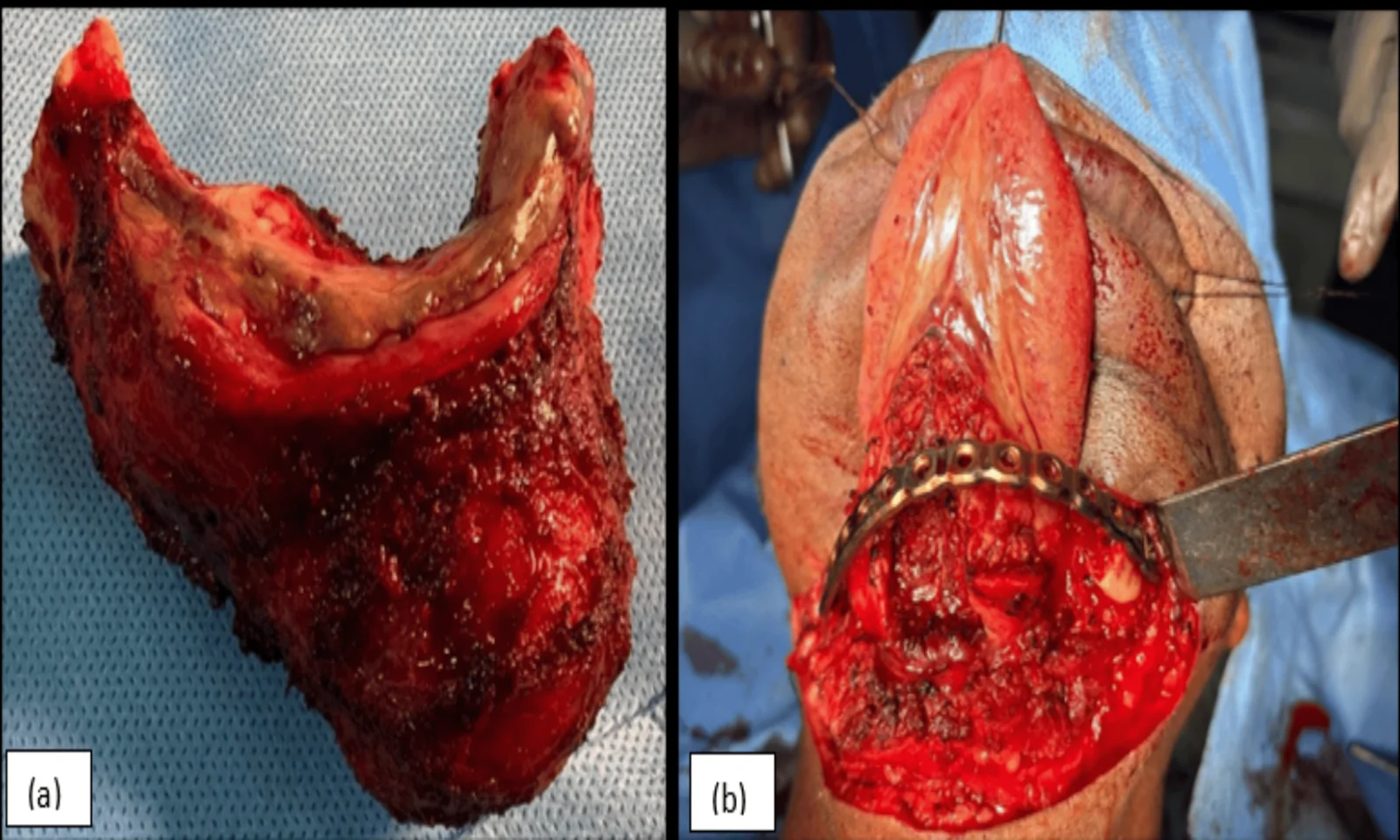
(a) Image showing excised specimen; (b) primary reconstruction procedure carried out using a 2.5 mm reconstruction plate following segmental mandibular osteotomy.

Defect reconstruction was achieved using a vascularised free fibula osteomyocutaneous flap. The lower extremity donor site was dissected (Figure 7a) to harvest a segment of the fibula along with its peroneal vascular pedicle and a matching skin paddle designed for intraoral lining (Figure 7b). A 10-cm segment of fibula was harvested and osteotomised. Two osteotomies were performed in the harvested fibular segment to facilitate appropriate adaptation to the mandibular defect and to reproduce the required mandibular curvature. The segment was then secured to the reconstruction plate (Figure 7c). Meticulous microvascular anastomosis was performed to re-establish perfusion. The peroneal artery and vena comitans of the fibula flap were anastomosed end-to-end and end-to-side, respectively, to appropriate recipient vessels, including the facial artery, external jugular vein, and common facial vein (Figures 7d, 7e). These vessels were selected because of their suitable calibre and accessibility, making them useful recipient veins for venous anastomosis in this setting. Flap viability was confirmed intraoperatively by checking for bright-red bleeding and adequate capillary refill of the skin paddle. The intraoral defect was closed watertight using resorbable sutures to isolate the reconstruction from oral secretions. The neck dissection and submental incisions were closed in anatomical layers over negative-pressure surgical drains. Histopathological evaluation of the bilateral neck dissection specimens confirmed reactive hyperplasia with no evidence of malignancy or ameloblastic extension.

**Figure 7:**
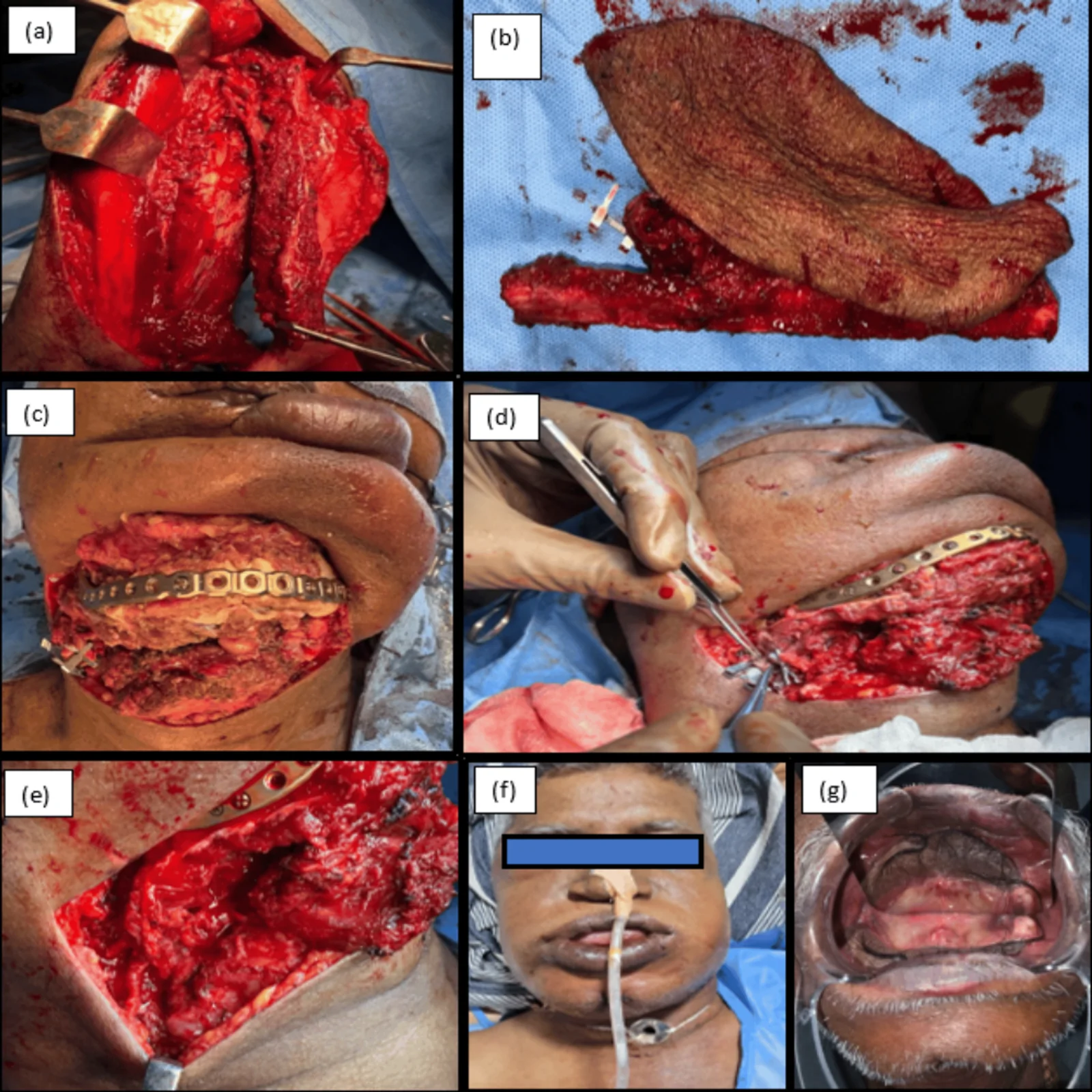
(a) Image showing harvesting of osteomyocutaneous fibula flap (b) Harvested vascularized Free Fibula Flap (c) Reconstruction plate fixed with the harvested free fibula flap (d),(e) Microvascular Anastomosis of Peroneal artery with facial artery and vena comitans with external jugular vein and common facial vein (f) Immediate post operative picture after tracheostomy (g)Intraoral image of reconstructed defect with free fibula flap at sixth month follow up

Given the extensive nature of the anterior mandibular resection, floor-of-mouth manipulation, and the risk of acute airway compromise from postoperative oedema, an immediate elective tracheostomy (Figure 7f) was performed. The patient tolerated the procedure well and was transferred to the surgical intensive care unit (ICU) for close monitoring of airway patency and microvascular flap protocols. At the six-month follow-up, the patient demonstrated satisfactory healing, good functional recovery, and acceptable facial symmetry (Figure 7g). The skin paddle appeared healthy and viable, with no evidence of flap compromise or clinical or radiographic signs of recurrence.

The patient remains under regular six-monthly clinical and radiographic surveillance, including panoramic radiography (OPG). Long-term follow-up is planned for a minimum of 10 years given the potential for late recurrence of ameloblastoma.

## Discussion

Ameloblastoma is a benign yet locally invasive epithelial odontogenic neoplasm that accounts for approximately 1% of oral tumours and cysts, showing a significant predilection for the posterior mandible but frequently causing massive expansile deformities when presenting in the anterior arch. Historically, the management of conventional (solid/multicystic) ameloblastomas has evolved significantly [1,4,6]; standard literature establishes that conservative modalities such as enucleation or curettage are plagued by unacceptably high recurrence rates ranging from 55% to 90% due to the tumour's micro-infiltrative growth pattern into surrounding cancellous bone [2,5,7]. Modern oncological protocols dictate that radical en bloc segmental resection with a 1.0-1.5 cm safe bony margin is the gold standard, effectively reducing recurrence rates to less than 15% [5,7,9]. To safely expose an extensive tumour involving the mandibular symphysis while avoiding the high aesthetic morbidity and long-term functional deficits of a midline lip-split incision, utilising a visor flap via a pull-through approach offers an excellent surgical pathway. While conventional ameloblastomas are classically considered benign with an extremely low rate of metastasis [7,10], elective selective neck dissection encompassing levels Ia, Ib, IIa, and IIb is an essential diagnostic and therapeutic consideration when there is clinical or radiological evidence of cortical perforation, secondary infection, or reactive lymphadenopathy that mimics malignant regional disease [5,7].

From a microscopic standpoint, a definitive diagnosis of conventional ameloblastoma relies on identifying classic histomorphological patterns, with the follicular and plexiform variants being the most prevalent [3,6]. The plexiform pattern, by contrast, presents as an anastomosing network or lace-like arrangement of epithelial cords bordered by similar polarised columnar cells. Furthermore, contemporary molecular oncology has heavily linked the pathogenesis of these jaw lesions to mutations in the mitogen-activated protein kinase (MAPK) signalling pathway, with the BRAF V600E mutation identified in up to 80% of mandibular cases [3,7]. While advanced immunohistochemical (IHC) profiling for the VE-1 antibody can be used to target this mutation, a definitive diagnosis in our case rested securely on the classic, highly characteristic histomorphological architecture. Following wide structural ablation of the anterior mandible, immediate microvascular reconstruction is mandatory to prevent the "Andy Gump" deformity, which leads to severe retropositioning of the tongue, floor-of-mouth collapse, cosmetic disfigurement, and complete loss of deglutition and speech articulation [8,11]. The vascularised free fibula osteocutaneous flap remains the premier choice for reconstructing extensive anterior mandibular gaps; its dense, bicortical bone stock provides excellent structural rigidity, easily tolerates multiple precise closing-wedge osteotomies to replicate the contour of the anterior mandibular arch, and provides a reliable peroneal vascular pedicle for microvascular anastomosis to recipient cervical vessels [8,12]. A critical technical refinement highlighted in this workflow is the technique of pre-bending the 2.5-mm titanium reconstruction plate along the native lower border of the mandible prior to executing the osteotomy cuts. This step acts as a perfect structural blueprint, ensuring that the original maxillo-mandibular relationships, vertical dimension of occlusion, and native condylar position within the glenoid fossae are accurately preserved and re-established post-resection [8]. Finally, given the extensive disruption of the suprahyoid musculature, bilateral manipulation of the neck during dissection, and the predictable onset of severe upper airway oedema, securing the airway via an immediate elective tracheostomy is a fundamental postoperative safety protocol [8].

## Conclusions

This case is noteworthy because of the unusually extensive involvement of the anterior mandible crossing the midline for an ameloblastoma, producing significant cortical expansion and destruction of the symphysis and parasymphysis regions while still presenting with relatively mild symptoms despite a long duration of two years. The surgical management was particularly challenging due to the need for adequate oncologic clearance while preserving important vascular structures and maintaining mandibular continuity and facial aesthetics. Successful treatment was achieved through segmental mandibular resection followed by immediate reconstruction with a reconstruction plate using a visor flap pull-through approach, which provided excellent surgical access with an improved aesthetic outcome and reduced risk of nerve injury. This approach represents a feasible option for achieving adequate surgical exposure and immediate microvascular reconstruction in extensive mandibular defects. Although no recurrence was evident at six months, long-term surveillance is essential, as in our case the patient is being followed at six-month intervals with a planned follow-up period of 10 years to assess for late recurrence and long-term oncological outcomes. This case emphasises the importance of early diagnosis, thorough radiographic evaluation, histopathological confirmation, and meticulous surgical planning in the management of large ameloblastomas to achieve favourable functional and cosmetic outcomes with a reduced risk of recurrence.
